# HOPX functions as a tumour suppressor in head and neck cancer

**DOI:** 10.1038/srep38758

**Published:** 2016-12-09

**Authors:** Lee Fah Yap, Sook Ling Lai, Sathya Narayanan Patmanathan, Ravindran Gokulan, C. Max Robinson, Joe B. White, San Jiun Chai, Pathmanathan Rajadurai, Narayanan Prepageran, Yew Toong Liew, Victor Lopes, Wenbin Wei, Robert J. Hollows, Paul G. Murray, Daniel W. Lambert, Keith D. Hunter, Ian C. Paterson

**Affiliations:** 1 Department of Oral and Craniofacial Sciences and Oral Cancer Research and Coordinating Centre, Faculty of Dentistry, University of Malaya, 50603 Kuala Lumpur, Malaysia; 2 Centre for Oral Health Research, Newcastle University, Newcastle, NE2 4BW, United Kingdom; 3 Unit of Oral and Maxillofacial Pathology, School of Clinical Dentistry, University of Sheffield, Sheffield, S10 2TA, Unite Kingdom; 4 Cancer Research Malaysia, Selangor, 47500 Subang Jaya, Malaysia; 5 Subang Jaya Medical Centre, 47500 Subang Jaya, Malaysia; 6 Department of Otorhinolaryngology, Faculty of Medicine, University of Malaya, 50603 Kuala Lumpur, Malaysia; 7 Department of Oral surgery, Edinburgh Postgraduate Dental Institute, University of Edinburgh, Edinburgh, EH3 9HA, United Kingdom; 8 Institute of Cancer and Genomic Studies, University of Birmingham, Birmingham, B15 2TT, United Kingdom; 9 Sheffield Institute of Translational Neuroscience, University of Sheffield, Sheffield, S10 2HQ, United Kingdom

## Abstract

Head and neck squamous cell carcinoma (HNSCC) is generalized term that encompasses a diverse group of cancers that includes tumours of the oral cavity (OSCC), oropharynx (OPSCC) and nasopharynx (NPC). Genetic alterations that are common to all HNSCC types are likely to be important for squamous carcinogenesis. In this study, we have investigated the role of the homeodomain-only homeobox gene, HOPX, in the pathogenesis of HNSCC. We show that HOPX mRNA levels are reduced in OSCC and NPC cell lines and tissues and there is a general reduction of HOPX protein expression in these tumours and OPSCCs. HOPX promoter methylation was observed in a subset of HNSCCs and was associated with a worse overall survival in HPV negative tumours. RNAseq analysis of OSCC cells transfected with HOPX revealed a widespread deregulation of the transcription of genes related to epithelial homeostasis and ectopic over-expression of HOPX in OSCC and NPC cells inhibited cell proliferation, plating efficiency and migration, and enhanced sensitivity to UVA-induced apoptosis. Our results demonstrate that HOPX functions as a tumour suppressor in HNSCC and suggest a central role for HOPX in suppressing epithelial carcinogenesis.

Squamous cell carcinomas (SCCs) that develop in the head and neck region (HNSCC) include cancers of the oral cavity, oropharynx and nasopharynx, which are tumours with different and distinct etiologies. Both oral squamous cell carcinoma (OSCC) and oropharyngeal carcinoma (OPSCC) are caused primarily by tobacco and alcohol, but there is now strong evidence implicating human papillomavirus with a sub-set of OPSCCs^1^,^2^. Nasopharyngeal carcinoma (NPC) is strongly associated with Epstein-Barr virus (EBV) infection^3^. Whilst there is some overlap in the profile of molecular alterations detected in the three tumour types, significant differences have been reported. For example, p53 mutations are common in OSCCs, but are less frequent in HPV-positive OPSCCs (compared with HPV-negative cases) and NPCs^4^. Genes that are commonly mutated and/or de-regulated in OSCC, OPSCC and NPC, therefore, are likely to be of fundamental importance to the development and progression of SCCs in general.

The homeodomain only protein, HOPX (also known as HOP, NECC1, LAGY or OB1), was initially identified as a gene essential for cardiac development^5^. HOPX is unusual because although it forms a classical homeodomain fold, it lacks several key DNA-binding residues that are conserved among other homeodomain proteins^5^,^6^,^7^. Rather than binding to DNA, two distinct regions on the surface of the HOPX protein are required for its ability to interact with other proteins such as serum response factor (SRF) and HDACs to modulate transcription^6^. There are three reported splice variants of the HOPX gene (HOPX-α, HOPX-β and HOPX-γ) that code for the same protein^8^. However, recent analysis of NCBI reference sequences indicates that there are five transcripts that encode three different proteins^9^, although the expression of these transcripts in different tissues has not yet been examined. The HOPX-β promoter contains CpG islands that are methylated in various cancers leading to down-regulation of HOPX expression, strongly suggesting that HOPX functions as a tumour suppressor^8^,^10^,^11^.

Our previously published microarray data^12^,^13^,^14^,^15^ showed that HOPX mRNA levels were reduced in both OSCC and NPC compared to their respective non-malignant controls. In the present study, we have extended these observations and show for the first time that the expression of HOPX is markedly down-regulated in three different subtypes of HNSCC, namely OSCC, NPC and OPSCC. Analysis of The Cancer Genome Atlas (TCGA) HNSCC dataset showed that hypermethylation of the HOPX-β promoter occurs in a sub-set of HNSCCs and this was associated with worse overall survival in HPV negative HNSCCs. Ectopic expression of HOPX in OSCC cells revealed that HOPX loss was associated with the deregulated transcription of genes involved in epithelial homeostasis. Additionally, ectopic expression of HOPX in both OSCC and NPC-derived cell lines inhibited cell proliferation, plating efficiency and migration, and enhanced sensitivity to UVA-induced apoptosis. Our results point to a central role for HOPX in suppressing epithelial carcinogenesis.

## Results

### Down-regulation of HOPX mRNA expression in OSCC and NPC

**Figure 1:**
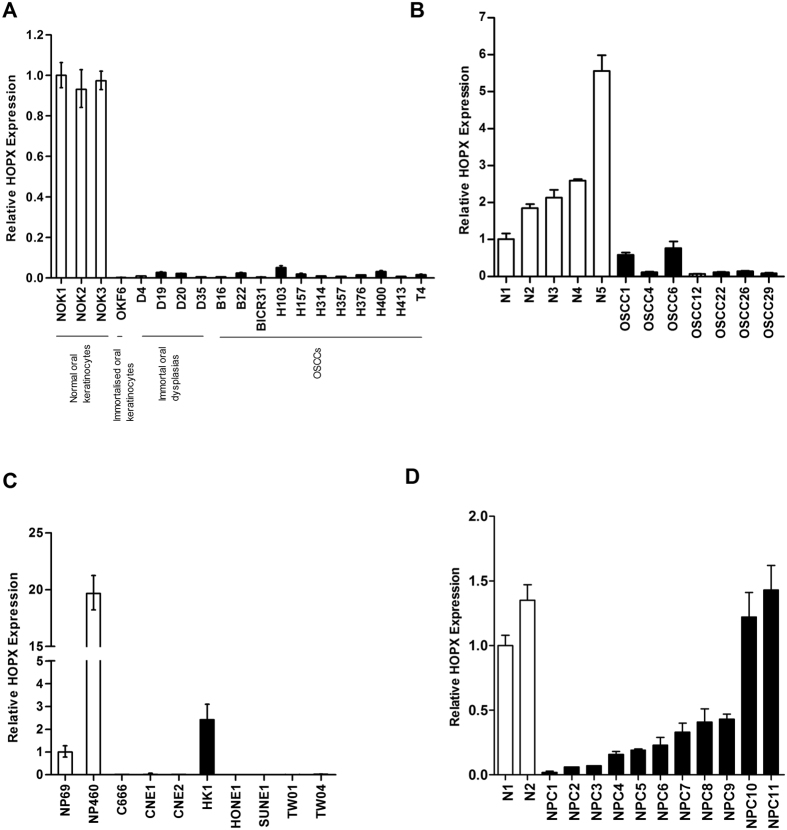
HOPX mRNA expression in OSCC and NPC. The expression of HOPX was examined by RT-qPCR in normal oral keratinocytes (NOK), immortalised oral keratinocytes (OKF6), together with cell lines derived from dysplasias (D4, D19, D20, D35) and OSCCs (**A**) and in tissue samples of normal oral mucosa and OSCCs (**B**). A similar down-regulation of HOPX expression was seen in NPC cell lines (**C**) and tissues (**D**). Experiments were performed in triplicate and error bars indicate standard deviations.

A re-examination of our previous microarray data^12^,^13^,^14^,^15^ demonstrated down-regulation of HOPX in both OSCC and NPC (Table 1). To validate these data, RT-qPCR analyses were performed to determine the mRNA levels of HOPX in a series of OSCC and NPC cell lines and tissues. In OSCC, compared to three cultures of normal oral keratinocytes, HOPX expression was markedly reduced in immortalized oral keratinocytes (n = 1), immortal cell lines derived from oral dysplasia tissues (n = 4) and SCCs (n = 11; Fig. 1A). HOPX mRNA levels were also significantly (p < 0.05) reduced in 6 of 7 primary OSCC tissues examined compared to five samples of normal oral mucosa (Fig. 1B).

Similar results were observed in NPC. HOPX mRNA levels were decreased in seven of eight NPC cell lines compared to the immortalized nasopharyngeal cell lines NP69 (Fig. 1C). HOPX expression was also significantly (p < 0.05) lower in 9 of 11 primary NPC tissues when compared to two samples of non-malignant nasopharyngeal epithelium (Fig. 1D).

### HOPX protein levels are reduced in primary HNSCCs

**Figure 2:**
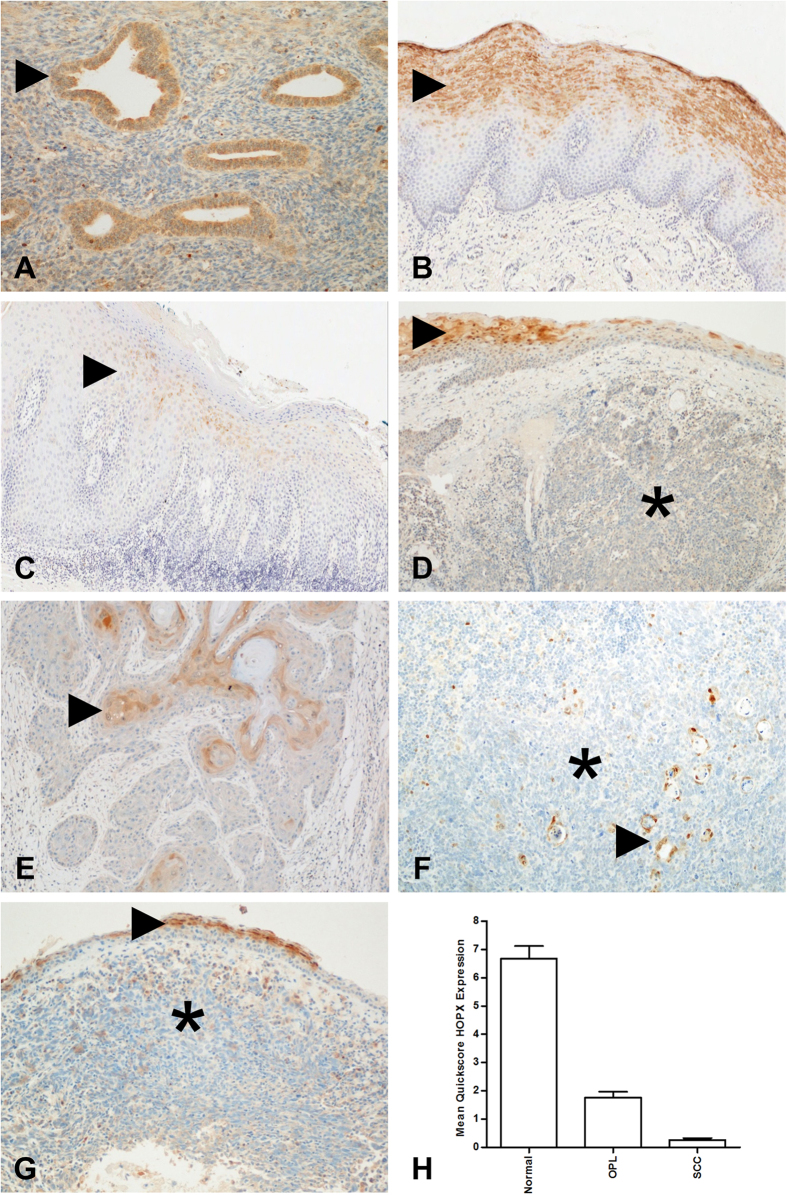
HOPX protein expression in HNSCCs by immunohistochemistry. The glands of secretory phase endometrium are known to express HOPX and were used as a positive control (**A**, arrowhead = gland). HOPX staining was strong in the keratinising layer of normal oral squamous epithelium (**B**,**D** and **G**, arrowhead = keratinising layer). By contrast, HOPX expression was reduced in oral premalignant lesions (**C**, arrowhead = keratinising layer). HOPX was absent in the majority of HNSCCs including OSCC, (**D**, * = malignant cells), OPSCC (**F**, * = malignant cells) and NPC (**G**, * = malignant cells). Focal HOPX staining co-localised with areas of keratinisation within tumours (**E** and **F**, arrowhead = keratinisation). Quantitation of HOPX expression using a quickscore method confirmed lower protein expression in premalignant lesions and OSCCs compared to normal oral squamous epithelium (**H**). Note: photomicrographs A, D-G HOPX Abcam ab57832, **B** and **C** HOPX Santa Cruz sc-30216. Experiment H used HOPX Santa Cruz sc-30216.

HOPX was strongly expressed in the glands of secretory phase endometrium (Fig. 2A) and in the keratinising layers of normal oral squamous epithelium (Fig. 2B). By contrast, HOPX expression was reduced in oral premalignant lesions (Fig. 2C). The majority of OSCCs (13 of 20) showed focal HOPX staining that co-localised with areas of keratinisation within the tumour (Fig. 2E), the remaining seven cases did not show any HOPX expression (Fig. 2D). The majority (19 of 27) OPSCCs showed focal weak HOPX staining (Fig. 2F), whilst the remaining eight cases did not show any HOPX expression. There was no correlation between HOPX expression and HPV status in this small series of OPSCCs (15 HPV positive, 12 HPV negative). Twenty NPCs were tested and all were HOPX negative (Fig. 2G). HOPX immunohistochemistry was carried out on a tissue microarray constructed from cores from normal oral mucosa (n = 31), oral premalignant lesions (n = 10) and from the invasive front of OSCCs (n = 42). HOPX expression was highest in normal oral squamous epithelium and reduced in oral premalignant lesions and OSCCs (p < 0.001; Fig. 2H).

### HOPX-β promoter methylation in HNSCC cell lines

**Figure 3:**
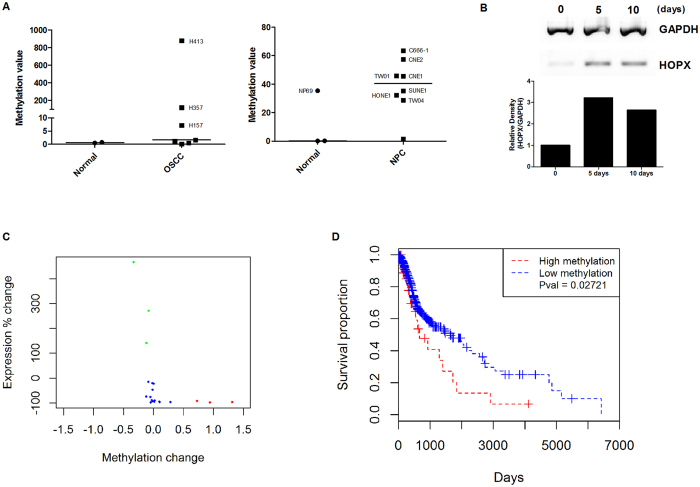
HOPX promoter methylation in HNSCC. Quantitative analysis of HOPX-β promoter methylation in OSCC and NPC cell lines with normal oral keratinocytes and immortalised nasopharyngeal epithelial cell lines (**A**). Treatment of H413 OSCC cells with the demethylating drug, zebularine, for 5–10 days induced re-expression of HOPX (**B**). HOPX-β promoter methylation was associated with a reduction of HOPX expression as determined by comparing expression in matched tumour and normal data within the TCGA HNSCC dataset (**C**). Hypermethylated cases are shown in red, those with higher expression than their matched normal are shown in green, with the remainder shown in blue. Kaplan Meier survival analysis showed there was a significant association between HOPX-β promoter methylation and worse overall survival (**D**).

Q-MSP analysis was carried out to assess the HOPX-β promoter methylation in a panel of 7 OSCC and 8 NPC cell lines, together with 2 cultures of normal oral keratinocytes and 3 immortalized nasopharyngeal epithelial cell lines (NP69, NP460, NP550). Three OSCC cell lines showed high methylation values, namely H413 (880), H357 (120) and H400 (7), while the two normal oral keratinocytes had methylation values less than 1 (Fig. 3A). Among the immortalised nasopharyngeal cell lines, NP69 showed the highest methylation value (35) while the other two normal nasopharyngeal cell lines studied (NP460 and NP550) showed values less than 0. Four NPC cell lines showed methylation values higher than 35, i.e. C666 (63), CNE2 (57), CNE1 (46) and TWO1 (46) (Fig. 3A). In support of these data, the expression of HOPX mRNA was re-activated in H413 cells (which showed the highest methylation value) following the treatment with the demethylating drug, Zebularine (Fig. 3B).

### HOPX-β expression and promoter methylation in primary HNSCC

There are 26 CpG sites on the Illumina^®^ HumanMethylation450 BeadChip (“450 k platform”) which are within the HOPX gene. Comparison of the DNA sequence information for the relevant probes with sequence information for the promoter region of the HOPX-β transcript, revealed that three of the 26 CpG sites (cg21899596, cg24852548 and cg01330221) were within the promoter region.

To obtain a measure of the total methylation of the promoter region, total methylation (beta) value across the three relevant CpG sites was calculated. For each site the beta value is a number between 0 (totally unmethylated) and 1 (100% methylated), so the sum of the three is a number between 0 (all sites totally unmethylated) and 3 (all sites 100% methylated). We chose an arbitrary cut off value of 1 to represent hyper-methylation across the three probes. Using these criteria, 53 out of 503 HNSCC tumours within the TCGA dataset showed hyper-methylation of the HOPX-β promoter. Of the 20 cases for which methylation and expression were available for both the tumour and matched normal tissues, HOPX was lower in 17 (85%) of cases; 3 of the 17 tumours showed evidence of HOPX-β promoter hyper-methylation and this was associated with a significant reduction of expression (Fig. 3C). Interestingly, HOPX expression was more than 100% higher in each of the remaining three tumours compared to matched normal controls.

Based on a univariate analysis of all the tumour samples, there was no statistically significant difference in overall survival between cases with and without HOPX-β promoter hyper-methylation. However, when HPV-ve cases were considered in isolation, 42 out of 407 tumours showed HOPX-β promoter hyper-methylation and there was a statistically significant association with worse overall survival (Fig. 3D).

### HOPX regulates the transcription of genes associated with tissue homeostasis

**Figure 4:**
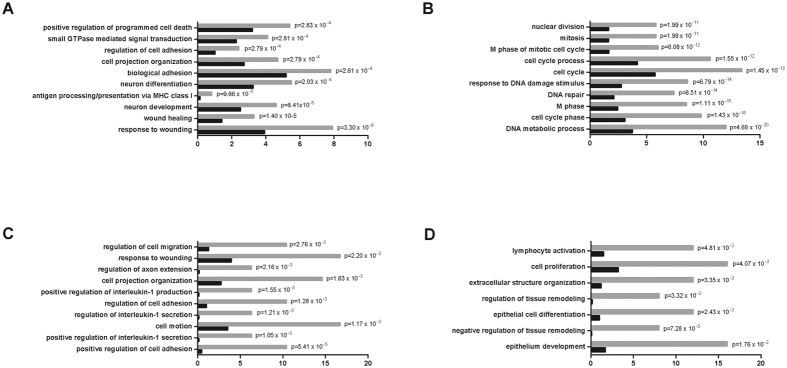
HOPX regulates the expression of genes involved in epithelial homeostasis. Gene ontology (GO) analysis of down-regulated (**A**) and up-regulated (**B**) genes identified by RNASeq following ectopic expression of HOPX in H376 OSCC cells. Genes with over 1.5 fold change in expression in H376/HOPX cells compared to H376-pIRES were subjected to GO analysis. A similar GO analysis was performed on those genes down-regulated by HOPX in H376 and up-regulated in primary OSCCs (**C**) and those up-regulated by HOPX in H376 and down-regulated in tumours (**D**). Genes altered in H376/HOPX were compared with genes whose expression was altered in micro-dissected OSCCs (GSE35261 dataset^16^).

To investigate the consequences of HOPX down-regulation in HNSCC, we analysed gene expression by performing RNAseq in H376 OSCC cells stably transfected with HOPX isoform b (H376/HOPX). Over-expression of HOPX in transfected H376 cells relative to vector only controls (H376/pIRES) was confirmed by both RT-qPCR, Western blot and immunocytochemical analyses (Figure S1). Following ectopic re-expression of HOPX in H376, 1525 genes were down-regulated (Table S1) and 995 up-regulated (Table S2) with an absolute fold change >1.5 and p value < 0.01. Gene ontology analysis revealed an enrichment of genes associated with cell proliferation, adhesion, cell death and response to DNA damage, (Fig. 4A and B). To examine the potential functional significance of HOPX loss in HNSCCs, we performed gene ontology analysis of genes regulated by HOPX in H376 that were also altered in primary tumours. To do this, we identified genes that were down-regulated by HOPX in H376 but up-regulated in micro-dissected OSCCs and vice versa (GSE35261 dataset^16^). Gene ontology analyses on these genes identified pathways associated with cell proliferation, adhesion and migration, as well as pathways that regulate epithelial development and differentiation (Fig. 4C and D). Collectively, these data indicate that HOPX functions to regulate squamous epithelial homeostasis and these mechanisms are altered in primary OSCCs.

### HOPX inhibits proliferation and migration and enhances the sensitivity of OSCC and NPC cells to DNA damage

Having shown that HOPX influences key pathways associated with homeostasis and carcinogenesis, we next tested these observations *in vitro*. Cell proliferation, migration and the response to DNA damage were examined in H376/HOPX cells and also in the NPC-derived cell line, HONE-1, stably transfected with HOPX (Figure S1).

**Figure 5:**
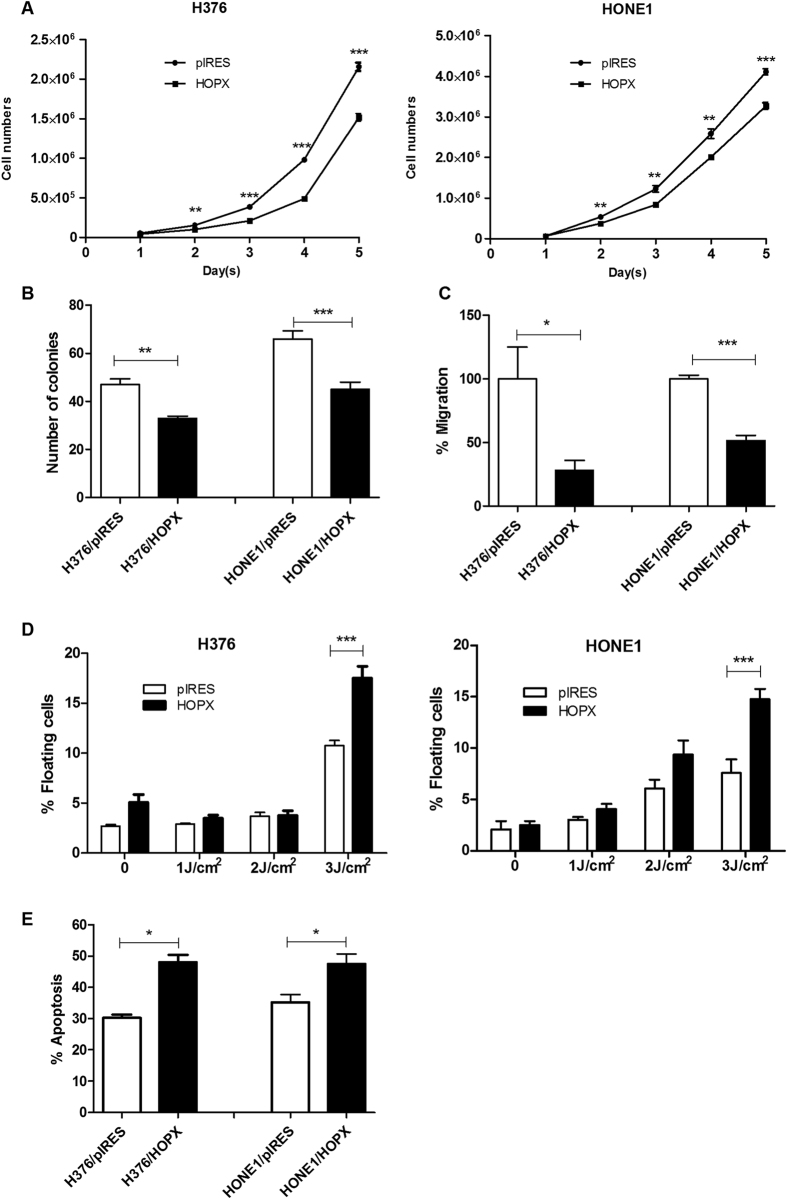
Tumour suppressive effects of HOPX in OSCC and NPC cells. The proliferation (**A**), plating efficiency (**B**) and migration (**C**) of HOPX expressing H376 OSCC and HONE1 NPC cells was significantly reduced compared to vector transfected controls. HOPX expressing H376 and HONE1 cells were more sensitive to DNA damage induced by UVA irradiation as determined by the percentage of floating cells in the culture dish (**D**) after 24 hours and enhanced in apoptosis as determined by flow cytometric analysis of Annexin V staining 24 hours after irradiation (3 J/cm^2^ UVA; E). Results are shown as mean ± SD values of triplicates. *ρ < 0.05, **ρ < 0.01 and ***ρ < 0.001.

Over-expression of HOPX resulted in a significantly slower growth rate (p < 0.01; Fig. 5A) and a reduced plating efficiency (p < 0.01; Fig. 5B) in both cell lines compared to their respective controls. LPA can induce SRF whose activity is inhibited by HOPX^17^ and we have previously shown that LPA is a motility factor for both OSCC and NPC cells (unpublished data;^18^). Therefore, we next investigated whether HOPX is involved in LPA-mediated migration and found that the migration of both HOPX-expressing H376 and HONE1 cells was significantly reduced compared to the vector controls (p < 0.05; Fig. 5C).

Next, we examined whether over-expression of HOPX influences the cellular response to UV-induced DNA damage. Following UVA exposure at 3 J/cm^2^, the number of floating (apoptotic) cells were significantly higher in both H376 and HONE1 cells expressing HOPX compared to vector controls (p < 0.001; Fig. 5D). These results were confirmed using flow cytometric analysis which showed an increase in Annexin V-positive cell populations in both HOPX-expressing H376 and HONE1 cells (p < 0.05; Fig. 5E), indicating that HOPX enhanced the sensitivity of these cells to UV-induced apoptosis.

## Discussion

OSCC, OPSCC and NPC are squamous cancers of the head and neck region with diverse aetiologies. Identification of genetic abnormalities that are common to all three cancer types might reveal genes/pathways fundamental to squamous carcinogenesis and lead to the identification of novel therapeutic targets. In the present study, we demonstrate that the expression of HOPX protein is consistently down-regulated in different sub-types of HNSCC when compared to their respective non-malignant epithelium *in vitro* and *in vivo*. Our data extend an early report showing loss of HOPX mRNA in another subtype of HNSCC, hypopharyngeal carcinoma^19^, and implicate HOPX as a universal tumour suppressor gene relevant to head and neck carcinogenesis. The decrease in HOPX expression in immortal dysplasia cells and tissue samples of oral premalignant lesions observed here suggests HOPX down-regulation occurs early during disease progression.

The loss of HOPX expression in other malignancies, such as gastric, pancreatic, esophageal, colorectal and lung cancers, is attributed to the hypermethylation of HOPX-β promoter^8^,^10^,^11^,^20^,^21^. In the present study, we show that the HOPX-β promoter is hypermethylated in approximately 50% of OSCC and NPC cell lines and that treatment of an OSCC cell line with a demethylating drug restored HOPX expression, suggesting that promoter methylation is in part responsible for the loss of HOPX expression in HNSCCs. Interrogation of the level 2 methylation data from the TCGA HNSCC dataset indicates that approximately 10% of tumours show evidence of HOPX-β promoter hypermethylation. Interestingly, this methylation correlated with a worse overall survival in HPV negative HNSCCs, which supports previous studies showing an association between HOPX-β promoter hypermethylation and patient prognosis^8^,^21^. It is noteworthy that we identified the five recently reported HOPX isoforms (that code for protein isoforms a, b and c)^9^ plus two apparently non-coding transcripts in the TCGA HNSCC RNASeq data. Clearly, the biological functions of HOPX in human tissues are more complex than previously thought and studies to elucidate the function of the HOPX isoforms, together with the identification of isoform- and tissue-specific binding partners are warranted.

To investigate the function of HOPX in the pathogenesis of HNSCC, we performed RNAseq on OSCC cells transfected with HOPX (isoform b). GO analyses of the transcriptionally altered genes indicated an enrichment of genes associated with cell proliferation, adhesion, cell death and response to DNA damage. Rather than validate a limited number of the transcriptional changes *in vitro*, we adopted an approach to identify pathways that were functionally relevant in tumours. To do this we performed GO analyses on those genes that were altered following HOPX expression in OSCC cells *in vitro* that were also transcriptionally deregulated in micro-dissected primary OSCCs^16^. Similarly, pathways related to cell proliferation, adhesion and migration were identified plus pathways associated with epithelial development and differentiation. Collectively, our results demonstrate that HOPX regulates the transcription of genes associated with epithelial homeostasis. Whilst it will be important to confirm these findings in normal keratinocytes following knockdown of endogenous HOPX, our data are entirely consistent with observations that HOPX regulates the balance between proliferation and differentiation in a number of cell types and controls late terminal differentiation in keratinocytes^9^,^22^.

To examine whether the pathways identified by GO analyses in the present study translated to a functional effect of HOPX expression on cell behaviour, we determined the effect of ectopic over-expression of HOPX in OSCC and NPC cells. Expression of HOPX in both OSCC and NPC cells inhibited cell proliferation and plating efficiency, similar to previous reports in different cancer types^5^,^8^,^10^,^11^,^20^,^23^. HOPX expression in OSCC and NPC cells also inhibited cell migration towards LPA. These observations can be explained by the fact that HOPX inhibits SRF activity, LPA has been shown to activate SRF^17^ and that SRF promotes migration and proliferation of gastric epithelial cells^24^. Consistent with this, HOPX has been shown to suppress c-fos transcription in a SRF dependent manner in endometrial and breast cancer cell lines^25^. These results are particularly noteworthy because we have recently observed that LPA signalling is aberrantly activated in NPC^18^. In addition, we found that re-expression of HOPX sensitised OSCC and NPC cells to UVA-induced apoptosis, confirming a functional role for HOPX in mediating the response to DNA damage. In contrast to a recent study in lung cancer^20^, we did not observe any induction of senescence following HOPX expression (data not shown). In the present study, we did not observe any alteration in the expression of differentiation markers (CK1, CK4, CK13 and involucrin) in HOPX-expressing OSCC cells (data not shown), which probably reflects a block in terminal differentiation in SCCs. However, HOPX was expressed in the surface keratinising layers of normal oral epithelium and HOPX expression co-localised with areas of keratinisation in OSCCs, data that are entirely consistent with HOPX being a regulator of late keratinocyte differentiation and that HOPX expression correlates with tumour keratinisation in epidermal SCCs^22^. Taken together, our data show that transcriptional regulation by HOPX directly affects tumour cell behaviour and that HOPX functions as a tumour suppressor in HNSCC.

In summary, we show that HOPX is consistently lost in OSCC, OPSCC and NPC, three distinct subtypes of HNSCC, which results in a more aggressive phenotype. Our results highlight a central role for HOPX as regulator of epithelial homeostasis and a suppressor of squamous carcinogenesis.

## Methods

### Cell lines and tissue samples

Details and culture conditions for the normal, dysplastic and malignant oral keratinocytes have been described previously^26^,^27^,^28^,^29^. The nasopharyngeal cell lines used in this study were immortalized nasopharyngeal epithelial cell lines (NP69, NP460, NP550) and NPC-derived cell lines (CNE1, HK1, HONE1, SUNE1, TW01, TW04, C666.1) which were a kind gift from Professor S.W. Tsao (Department of Anatomy, University of Hong Kong, Hong Kong, China). In experiments to examine the effect of demethylation on the expression of HOPX, cells were treated with zebularine (200 μM in PBS) for up to 10 days. Media containing fresh zebularine were changed every 48 hours.

Archival tissue samples of OSCC, OPSCC and NPC, together with a set of tissue microarrays (TMA) comprising three cores from each of 31 normal oral mucosa, 10 oral premalignant lesions and 42 OSCC (cores taken from the invasive front) were used. Ethical approval for these studies were obtained from the UK National Research Ethics Service (References 08/SO709/70 and 11/NE/0118) and the Independent Ethics Committee, Sime Darby Healthcare, Malaysia (Ref. # 201206.2). Informed consent was not required by the ethics committees for this retrospective study of anonymized archived formalin-fixed paraffin embedded tissue samples (material surplus to diagnostic requirements). Fresh biopsy specimens of OSCC, NPC and normal oral mucosa were harvested with appropriate ethical approval from the South Birmingham Research Ethics Committee (0769) and the Medical Research Ethic Committee of the Ministry of Health Malaysia (KKM/NIHSEC/P13-494). Written informed consent was obtained from all participants. All methods were carried out in accordance with the approved guidelines. The NPCs used in this study were non-keratinising and EBER-positive.

### Reverse transcription PCR (RT-PCR) and reverse transcription quantitative PCR (RT-qPCR)

Total RNA was extracted using an RNeasy Mini Kit (Qiagen) and subjected to reverse transcription using oligo(dT) primer and Superscript III (Invitrogen). HOPX expression was examined by RT-PCR, using standard techniques. Primer sequences are given in Table S3.

Q-PCR was performed in triplicate using the ABI Prism 7000 Sequence Detection System and TaqMan Gene Expression Assays (HOPX: Hs04188695_m1; Applied Biosystems, USA). GAPDH was amplified in the same reaction to serve as a reference transcript for internal control for normalization. Fold changes in gene expression were measured using the comparative threshold cycle method (ΔΔCt).

### Immunohistochemistry

HOPX expression was determined in primary OSCC, NPC and OPSCC tissue samples by immunohistochemistry. Two primary antibodies (Abcam ab57832 [mouse monoclonal 1:100] and Santa Cruz sc-30216 [rabbit polyclonal 1:200]) were used as indicated and their specificities proven to be comparable by using secretory phase endometrium as a positive control^25^. The primary antibody was omitted from negative controls. For the TMA, HOPX staining was assessed using the modified Quickscore method^30^, with scoring of each core for extent of staining (0–3) and intensity of staining (0–3) by two independent assessors (KDH and JW). The results were multiplied and the mean score of 3 separate cores used in subsequent analyses.

### Quantitative methylation-specific PCR

Genomic DNA from cell lines was extracted using DNAEasy Blood and Tissue Kit (Qiagen) and 1 μg of DNA subjected to bisulfite treatment using EZ DNA Methylation-LightningTM Kit (Zymo Research, CA, United States). Quantitative Methylation-Specific PCR (Q-MSP) was performed to quantify the methylation level of HOPX- β promoter, using an EpiTect MethyLight PCR +ROX Vial Kit (Qiagen) and the 7500 Fast Real-Time PCR system (Applied Biosystems). The PCRs condition together with primer and probe sequences are shown in Table S3. Serial dilutions of methylated human control DNA (bisulfite converted; Qiagen) were used to construct the calibration curve. Methylated and unmethylated human control DNA (bisulfite converted; Qiagen) were used as controls. The methylation value was defined by a ratio of HOPX-β normalized to β-actin and then multiplied by 100, according to the comparative cycle threshold method^8^.

### HOPX-β promoter methylation analysis using The Cancer Genome Atlas HNSCC dataset

To investigate methylation of the HOPX-β transcript promoter region, data from The Cancer Genome Atlas (TCGA) using the “Data matrix” option within the TCGA data portal were downloaded. We downloaded all available level 2 methylation data which had been produced using the Illumina^®^ HumanMethylation450 BeadChip (“450 k platform”). The 450 k platform provides methylation measurements for over 485,000 CpG sites. The downloaded data contain methylated and unmethylated intensity values for each sample and CpG site. From these intensity values, the methylation (“beta”) values were calculated as the ratio:

methylated intensity/ (methylated intensity + unmethylated intensity)

Annotation information for the 450 k platform was obtained directly from Illumina’s website.

All available clinical data were downloaded in the “Biotab” format and level 3 RNA-sequencing data downloaded based on the Illumina HiSeq 2000 RNA Sequencing platform (Version 2). We used the files labelled “rsem.genes.normalized_results”, which contain normalised expression levels for over 20,000 genes.

There were 561 samples for which both methylation and clinical data were available (two metastatic tumour cases were excluded). Among these, 511 samples were from tumours, with the other 50 being from matched normal tissue samples. RNA-sequencing data were available for 503 of the 511 tumour samples and for 20 of the 50 matched normal samples.

All calculations were performed using the R programming language (R Core Team, 2015). Kaplan-Meier analyses with log-rank statistical tests of overall survival were performed using the “Survival” package. Tumour samples were categorised as “hyper-methylated” if their total beta values across all three and the relevant CpG sites exceeded 1.0. This threshold was chosen after consideration of the distribution of total beta values for all normal and tumour samples. A significance level of 5% was used in all statistical tests.

### Ectopic expression of HOPX

The coding region of HOPX was amplified from normal oral keratinocytes by RT-PCR using primers 5′AGGGACCATGTCGGCGGAGA3′ and 5′GCTGTCAATGCCTGCCATCT3′ and cloned in a sense orientation into the vector pIRES-neo2 (Takahara Bio Europe⁄Clontech Saint-Germainen-Laye, France). To establish cells stably overexpressing HOPX or the empty vector, H376 and HONE1 cells were transfected with 10 μg plasmid DNA using Fugene 6 transfection reagent (Roche, Burgess Hill, UK). Stable transfectants were selected in medium containing 400 μg/ml G418.

### RNA sequencing and bioinformatics

Total RNA was extracted from H376/HOPX and H376/pIRES in triplicate. Strand specific library preparation and paired-end RNA sequencing (Illumina HiSeq 4000) were performed by BGI Tech Solutions (Hong Kong) Co Ltd. Sequence reads were aligned to hg19 reference sequence using subread aligner and mapped sequencing reads were assigned to hg19 refGene genes using featureCounts. RefGene exon coordinates were obtained from the UCSC table browser. Gene symbol and description was obtained from the NCBI gene database. Genes with read count per million <1 in more than half of the samples were removed. The data was normalized using TMM (trimmed mean of M values) method. Differentially expressed genes were identified using edgeR.

To identify genes dysregulated in primary OSCC tumour cells compared with the normal control, the dataset, GSE35261, containing Oparray expression data from 33 laser-captured microdissected normal tissues, oral dysplastic lesions (ODLs), and invasive cancers from 11 patients were downloaded from Gene Expression Omnibus (GEO). The raw data was background subtracted using normexp method and normalized using quantile method of limma package. Differentially expressed probe sets were identified using limma (Smyth, 2004) with the criteria of absolute fold change >1.5 and limma p value < 0.01. Probe set annotation was obtained from GEO GPL8950 and reannotated according to NCBI gene database. For gene lists comparison, only one gene per probe set was used when two or more genes are associated with a probe set]. Gene ontology analysis was performed using The Database for Annotation, Visualization and Integrated Discovery (DAVID) vs 6.7.

### Cell proliferation and plating efficiency assays

2 × 10^4^ cells per dish were seeded into 60 mm dishes in triplicate. Cell proliferation was determined daily by trypsinising and counting on a CASY cell counter (Innovatis AG). For plating efficiency assays, single-cell suspensions of 100 cells per dish were seeded into 60-mm dishes and media were replenished every two days. After 14 days, the cells were fixed with 4% formaldehyde and stained with 0.1% crystal violet. A cluster of at least 50 cells under 20X magnification was considered as a colony and the numbers of colony in each dish were counted microscopically.

### Transwell migration assays

Migration assays were carried out using fibronectin-coated (10 μg/mL) polycarbonate filters (8 μm pore size, Transwell, Corning). Cells were cultured in growth medium/0.5% FBS overnight and treated with 10 μg/mL Mitomycin C (Merck Millipore) for two hours. After trypsinisation, cells were suspended in migration buffer [serum-free medium containing 0.25 mg/mL fatty acid-free human albumin (Sigma)]. 1 × 10^6^ cells were seeded into the upper chamber and allowed to migrate for 19 hours in the presence of 2.5 μM lysophosphatidic acid (LPA) in the lower chamber. Migrated cells were stained with 0.1% crystal violet and counted microscopically in five random fields under 20X magnification.

### UVA-induced cell death and apoptosis assays

3 × 10^5^ cells per dish were seeded into 60-mm dishes and incubated for 72 hours to reach 80% confluency. The cells were washed twice in phosphate-buffered saline (PBS) and subjected to UVA irradiation (3 J/cm^2^) using a UV DNA-crosslinker (UVGL-58 model, UVP, LLC, Upland, CA). The cells were then cultured in complete growth media for 24 hours. The media were subsequently centrifuged at 3000× g for 10 minutes to pellet the floating cells and the attached cells were collected by trypsinisation. Both floating and attached cells were counted using a haemocytometer. The percentage of cell death was calculated as the number of floating cells over the total cell number.

UVA-induced apoptosis was examined using an Annexin V: FITC apoptosis detection kit (BD Biosciences, San Jose, CA). Briefly, 24 hours post UVA irradiation, the floating cells were collected by centrifugation and attached cells treated with Accutase (BD, Innovative Cell Technologies) followed by centrifugation. Both floating and attached cells were pooled, washed twice with cold PBS and resuspended in 1X Binding Buffer. The cells were stained with FITC-conjugated Annexin V and PI and analyzed by a FACS Canto II flowcytometer (BD Bioscience, San Jose, CA). The amount of apoptosis was determined by expressing the total number of cells in early (Q2) and late (Q4) apoptosis as a percentage of the total number of cells (Q1–Q4).

## Additional Information

**How to cite this article:** Yap, L. F. *et al*. HOPX functions as a tumour suppressor in head and neck cancer. *Sci. Rep.* **6**, 38758; doi: 10.1038/srep38758 (2016).

**Publisher's note:** Springer Nature remains neutral with regard to jurisdictional claims in published maps and institutional affiliations.

## Supplementary Material

Supplementary Information

## Figures and Tables

**Table 1:** Down-regulation of HOPX mRNA in our previous microarray data.

Materials	Comparison	Analysis method	Results	References
OSCC cell lines	Mortal OSCC (n = 4) vs Immortal OSCC (n = 11)	SAM	>5-fold down-regulated in Immortal OSCCs	14
OSCC cell lines	Normal keratinocytes (n = 3) vs OSCC (n = 8)	SAM	4.8 fold down-regulated in OSCCs	15
NPC tissues	Non-malignant nasopharyngeal tissue (n = 3) vs NPC (n = 25)	GCOS pairwise comparison	Significant down-regulation in 8/25 (32%) of NPCs	12
NPC microdissected tissues	Normal epithelium (n = 3) vs NPC (n = 15)	GCOS pairwise comparison	Significant down-regulation in 9/15 (60%) of NPCs	13

SAM: Significance analysis of microarrays.

GCOS: GenChip Operating Software.
