# Supplementary material for: HOPX functions as a tumour suppressor in head and neck cancer
Source: Sci Rep. 2016 Dec 9;6:38758. doi: 10.1038/srep38758 (PMC5146930; doi:10.1038/srep38758)
Supplement: Supplementary Information [file srep38758-s1.pdf]

HOPX functions as a tumour suppressor in head and neck cancer

Lee Fah Yap, Sook Ling Lai, Sathya Narayanan Patmanathan, Ravindran Gokulan, C Max Robinson, Joe B White, San Jiun Chai, Pathmanathan Rajadurai, Wenbin Wei, Robert J Hollows, Paul G Murray, Daniel W Lambert, Keith D Hunter and Ian C Paterson

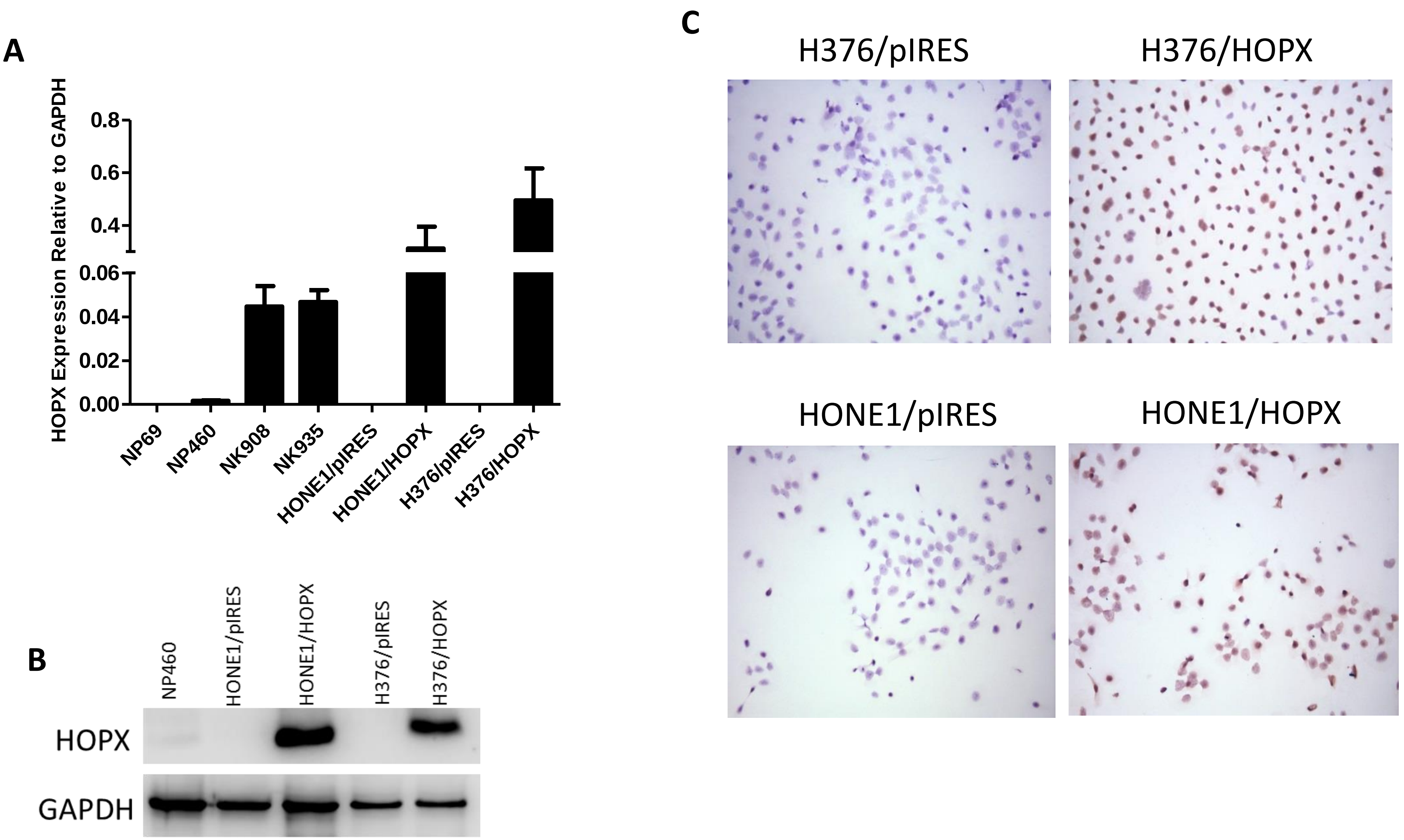

Figure S1. Confirmation of HOPX expression in H376 and HONE1 cells following stable transfection of HOPX cDNA. (A) mRNA expression in nasopharyngeal epithelial cells (NP69, NP460), normal oral keratinocytes (NK908, NK 935) and the transfected cells, as determined by RT-qPCR. (B) Western blot analysis of HOPX protein levels in NP460 and transfected cells. (C) HOPX expression in transfected cells and vector controls as detected by immunocytochemistry. Proteins were detected in B and C using the Santa Cruz sc-30216 antibody

Table S1. List of down-regulated in H376 cells transfected with HOPX. Top 100 genes based on fold change.

Symbol	description	PValue	Fold Change
LOC101929415	uncharacterized LOC101929415	6.44E-77	-165.225
FAM221A	family with sequence similarity 221, member A	1.29E-21	-67.5819
TP73-AS1	TP73 antisense RNA 1	6.82E-19	-52.4967
VSTM2L	V-set and transmembrane domain containing 2 like	7.91E-64	-33.6135
GAL	galanin/GMAP prepropeptide	1.01E-27	-28.2347
KRT19	keratin 19, type I	2E-277	-27.7507
FKBP10	FK506 binding protein 10, 65 kDa	1.3E-205	-24.2883
KRTAP2-3	keratin associated protein 2-3	1.01E-20	-21.0722
RNF182	ring finger protein 182	3.46E-20	-18.2854
SOX2	SRY (sex determining region Y)-box 2	4.25E-35	-17.0919
LCP1	lymphocyte cytosolic protein 1 (L-plastin)	6.5E-157	-16.6159
NGB	neuroglobin	7.87E-38	-10.603
COLCA2	colorectal cancer associated 2	9.3E-25	-10.2877
NACAD	NAC alpha domain containing	5.21E-46	-10.013
SCARF2	scavenger receptor class F, member 2	1.43E-12	-9.91398
TMOD1	tropomodulin 1	1.52E-27	-9.59575
KCNH1	potassium channel, voltage gated eag related subfamily H, member 1	1.97E-11	-9.18221
LYPD1	LY6/PLAUR domain containing 1	4.23E-17	-9.17353
CYP27A1	cytochrome P450, family 27, subfamily A, polypeptide 1	1.72E-18	-8.97221
GIPR	gastric inhibitory polypeptide receptor	5.49E-31	-8.641
N4BP2L1	NEDD4 binding protein 2-like 1	1.88E-18	-8.43861
CRIP1	cysteine-rich protein 1 (intestinal)	3.54E-57	-8.3578
ZFP3	ZFP3 zinc finger protein	1.43E-36	-8.19685
LSP1	lymphocyte-specific protein 1	1.54E-22	-7.98267
P2RY6	pyrimidinergic receptor P2Y, G-protein coupled, 6	3.09E-57	-7.98242

SEPP1	selenoprotein P, plasma, 1	6.54E-74	-7.94536
CCSER1	coiled-coil serine-rich protein 1	1.31E-20	-7.80882
MCOLN3	mucolipin 3	1.55E-15	-7.73652
SCARF1	scavenger receptor class F, member 1	3.32E-16	-7.52017
NSUN7	NOP2/Sun domain family, member 7	3.89E-18	-7.33462
SERPINA1	serpin peptidase inhibitor, clade A (alpha-1 antiproteinase, antitrypsin), member 1	6.2E-128	-7.27391
CGNL1	cingulin-like 1	3.24E-24	-7.21209
ADAM28	ADAM metallopeptidase domain 28	6.13E-35	-7.19047
H19	H19, imprinted maternally expressed transcript (non-protein coding)	7.89E-40	-6.98284
TMEM56	transmembrane protein 56	1.17E-40	-6.95241
BCHE	butyrylcholinesterase	2.06E-76	-6.88829
B4GALNT2	beta-1,4-N-acetyl-galactosaminyl transferase 2	2.19E-80	-6.83064
MEIS3P1	Meis homeobox 3 pseudogene 1	1.14E-11	-6.81425
KIF12	kinesin family member 12	7.51E-35	-6.8065
MEIS3	Meis homeobox 3	6.23E-43	-6.71411
HRAT92	heart tissue-associated transcript 92	2.94E-37	-6.66796
SLFN11	schlafen family member 11	7.35E-87	-6.62371
NRCAM	neuronal cell adhesion molecule	1.56E-63	-6.55182
JAZF1	JAZF zinc finger 1	9.4E-46	-6.5341
CADPS2	Ca++-dependent secretion activator 2	1.11E-22	-6.37956
TRPC1	transient receptor potential cation channel, subfamily C, member 1	1.79E-26	-6.35818
ELMO1	engulfment and cell motility 1	4.45E-59	-6.35
PRRT2	proline-rich transmembrane protein 2	3.76E-16	-6.26446
ACP5	acid phosphatase 5, tartrate resistant	3.65E-51	-6.23569
GMPR	guanosine monophosphate reductase	4.61E-27	-6.19317
BMP5	bone morphogenetic protein 5	1.58E-36	-6.19285
CFI	complement factor I	6.21E-42	-6.1735
ACSL5	acyl-CoA synthetase long-chain family member 5	3.43E-39	-6.15366
IGFBP4	insulin-like growth factor binding protein 4	1.99E-24	-6.09255

CALML3	calmodulin-like 3	2E-59	-6.06916
CRIP2	cysteine-rich protein 2	5.61E-67	-5.95039
SRRM3	serine/arginine repetitive matrix 3	8.2E-65	-5.93239
FAM127C	family with sequence similarity 127, member C	7.07E-16	-5.85449
COL8A2	collagen, type VIII, alpha 2	2.61E-19	-5.78648
AUTS2	autism susceptibility candidate 2	5.63E-22	-5.78074
PRR15	proline rich 15	1.82E-21	-5.63077
SEPT4	septin 4	5.37E-14	-5.54394
GAMT	guanidinoacetate N-methyltransferase	3.61E-21	-5.50358
CSF1R	colony stimulating factor 1 receptor	4.54E-32	-5.48911
FCHO1	FCH domain only 1	2.12E-10	-5.39925
RET	ret proto-oncogene	6.55E-10	-5.32676
WTIP	Wilms tumor 1 interacting protein	1.14E-33	-5.25544
PLEKHB1	pleckstrin homology domain containing, family B (evectins) member 1	9.47E-34	-5.17769
CLDN9	claudin 9	3.5E-21	-5.06952
ISYNA1	inositol-3-phosphate synthase 1	1.29E-56	-5.03941
CX3CL1	chemokine (C-X3-C motif) ligand 1	2.69E-63	-4.99636
C3orf18	chromosome 3 open reading frame 18	1.52E-11	-4.99144
FXYD6	FXYD domain containing ion transport regulator 6	9E-13	-4.95968
LOC100130673	phosphoribosyl pyrophosphate synthetase 2 pseudogene	8.8E-12	-4.87296
COL9A2	collagen, type IX, alpha 2	1.05E-50	-4.82754
ADGRB2	adhesion G protein-coupled receptor B2	3.39E-30	-4.81975
ETV7	ets variant 7	5.05E-11	-4.79312
SEMA3E	sema domain, immunoglobulin domain (Ig), short basic domain, secreted, (semaphorin) 3E	5.43E-35	-4.78048
PDGFRB	platelet-derived growth factor receptor, beta polypeptide	1.83E-08	-4.76702
VWCE	von Willebrand factor C and EGF domains	1.1E-08	-4.76512
TMEM221	transmembrane protein 221	1.64E-10	-4.73385
ANKRD1	ankyrin repeat domain 1 (cardiac muscle)	1.05E-15	-4.68865

SALL2	spalt-like transcription factor 2	3.81E-09	-4.6569
RASAL1	RAS protein activator like 1 (GAP1 like)	2.15E-13	-4.64514
C4orf26	chromosome 4 open reading frame 26	6.91E-08	-4.62201
RNF175	ring finger protein 175	1.27E-10	-4.58599
HMCN1	hemicentin 1	1.23E-10	-4.52683
OLFML2B	olfactomedin-like 2B	6.06E-12	-4.50986
C7orf65	chromosome 7 open reading frame 65	1.71E-12	-4.50049
TLCD2	TLC domain containing 2	2.26E-41	-4.49192
SCNN1B	sodium channel, non voltage gated 1 beta subunit	2.77E-22	-4.47437
WDR72	WD repeat domain 72	3.07E-11	-4.47228
RNF225	ring finger protein 225	8.12E-10	-4.47003
TMEM92	transmembrane protein 92	1.19E-07	-4.44328
NTNG1	netrin G1	1.35E-13	-4.37724
MPP1	membrane protein, palmitoylated 1, 55kDa	3.33E-41	-4.37465
SYK	spleen tyrosine kinase	1.31E-50	-4.35473
SYT17	synaptotagmin XVII	8.34E-10	-4.27938
COL22A1	collagen, type XXII, alpha 1	4.77E-16	-4.27592

Table S2. List of up-regulated in H376 cells transfected with HOPX. Top 100 genes based on fold change.

Symbol	description	PValue	Fold Change
ANXA10	annexin A10	1.82E-71	1053.516928
HOPX	HOP homeobox	0	800.5521021
CACNG8	calcium channel, voltage-dependent, gamma subunit 8	1.86E-23	225.3094123
LOC285629	uncharacterized LOC285629	6.27E-28	85.48355133
MDGA2	MAM domain containing glycosylphosphatidylinositol anchor 2	6.84E-23	23.70498301
WISP3	WNT1 inducible signaling pathway protein 3	1.69E-35	22.92882994
SPTSSB	serine palmitoyltransferase, small subunit B	3.75E-26	22.30730833
SFRP1	secreted frizzled-related protein 1	1.55E-95	21.47099248
ABCC9	ATP-binding cassette, sub-family C (CFTR/MRP), member 9	8.32E-36	21.01360621
S100P	S100 calcium binding protein P	1.49E-59	19.28078184
ROBO2	roundabout guidance receptor 2	1.93E-16	18.67934797
FBN2	fibrillin 2	2.5E-144	18.23549934
SHC4	SHC (Src homology 2 domain containing) family, member 4	2.74E-16	16.87978029
HSD17B2	hydroxysteroid (17-beta) dehydrogenase 2	3.9E-16	16.29814383
CA9	carbonic anhydrase IX	1.2E-151	14.94187511
HAS2	hyaluronan synthase 2	7.53E-52	14.18148189
TRIM31	tripartite motif containing 31	1.04E-18	13.66811128
SPINK6	serine peptidase inhibitor, Kazal type 6	1.09E-34	12.63526872
AOX1	aldehyde oxidase 1	1.72E-77	10.66649502
PI3	peptidase inhibitor 3, skin-derived	2.59E-92	10.32741782
SDC2	syndecan 2	2.71E-15	9.384561924
KCCAT211	renal clear cell carcinoma-associated transcript 211	2.15E-74	9.170796586
MGAM	maltase-glucoamylase	4.11E-96	9.07073963

BMP4	bone morphogenetic protein 4	8.2E-18	8.817985902
SEMA6D	sema domain, transmembrane domain (TM), and cytoplasmic domain, (semaphorin) 6D	1.36E-24	8.799605917
DEFB1	defensin, beta 1	4.71E-26	8.792669252
CCAT1	colon cancer associated transcript 1 (non-protein coding)	3.75E-46	8.558991529
FABP4	fatty acid binding protein 4, adipocyte	5.91E-35	8.549103531
DHRS9	dehydrogenase/reductase (SDR family) member 9	3.32E-11	8.42658643
PCSK5	proprotein convertase subtilisin/kexin type 5	1.57E-64	8.173659449
PID1	phosphotyrosine interaction domain containing 1	1.5E-31	8.155329867
CLCA4	chloride channel accessory 4	3.57E-15	7.909133974
SLC7A2	solute carrier family 7 (cationic amino acid transporter, y+ system), member 2	7.72E-61	7.786071541
OSBP2	oxysterol binding protein 2	1.4E-106	7.718485288
TENM1	teneurin transmembrane protein 1	1.04E-24	7.291230235
CD74	CD74 molecule, major histocompatibility complex, class II invariant chain	5.57E-53	7.086659317
MUC12	mucin 12, cell surface associated	2.24E-20	6.668999107
KRT14	keratin 14, type I	2.51E-37	6.340162712
CPS1	carbamoyl-phosphate synthase 1, mitochondrial	8.49E-26	6.328491409
TNFSF11	tumor necrosis factor (ligand) superfamily, member 11	7.28E-13	6.180249168
KRT34	keratin 34, type I	6.24E-73	6.114102939
DMBT1	deleted in malignant brain tumors 1	3.97E-79	6.006506855
SLC6A14	solute carrier family 6 (amino acid transporter), member 14	4.35E-13	5.767679941
AJAP1	adherens junctions associated protein 1	6.57E-49	5.65943183
FGFR1	fibroblast growth factor receptor 1	1.02E-13	5.601425905
PDE4B	phosphodiesterase 4B, cAMP-specific	1.16E-11	5.471686164
CDH19	cadherin 19, type 2	4.03E-13	5.445368184
LINC01234	long intergenic non-protein coding RNA 1234	7.76E-13	5.42729578
SASH1	SAM and SH3 domain containing 1	7.39E-23	5.267275879
PPFIBP2	PTPRF interacting protein, binding protein 2 (liprin beta 2)	1.54E-40	5.259277056
IFI27	interferon, alpha-inducible protein 27	2.4E-21	5.250737485

CPA4	carboxypeptidase A4	1.09E-09	5.188840326
FRMD3	FERM domain containing 3	6.29E-22	5.167818311
STEAP4	STEAP family member 4	1.41E-52	4.993322476
DPYSL2	dihydropyrimidinase-like 2	1.26E-40	4.981932355
LINC00707	long intergenic non-protein coding RNA 707	1.86E-28	4.976144844
LINC00052	long intergenic non-protein coding RNA 52	8.3E-29	4.96457157
A2ML1	alpha-2-macroglobulin-like 1	4.51E-50	4.9494145
PLTP	phospholipid transfer protein	3.71E-36	4.822372212
FOXI1	forkhead box I1	8.55E-34	4.698158159
UGT2B17	UDP glucuronosyltransferase 2 family, polypeptide B17	3.74E-12	4.576265298
RGMA	repulsive guidance molecule family member a	3.43E-08	4.565807336
SLC22A3	solute carrier family 22 (organic cation transporter), member 3	1.08E-10	4.52826627
CA12	carbonic anhydrase XII	1.33E-51	4.39720143
IDO1	indoleamine 2,3-dioxygenase 1	2.16E-08	4.397017654
DOCK8	dedicator of cytokinesis 8	1.07E-15	4.359722835
ABCG2	ATP-binding cassette, sub-family G (WHITE), member 2 (Junior blood group)	1.17E-08	4.313438743
SLC5A1	solute carrier family 5 (sodium/glucose cotransporter), member 1	1.64E-10	4.299830021
SCG5	secretogranin V	3.5E-38	4.297594092
TNF	tumor necrosis factor	1.5E-15	4.190088387
ADGRL2	adhesion G protein-coupled receptor L2	6.06E-16	4.145887697
KRT6B	keratin 6B, type II	1.47E-63	4.014312372
LOC152225	uncharacterized LOC152225	9.67E-11	3.985606728
GSDMC	gasdermin C	2.88E-17	3.984881461
MUC3A	mucin 3A, cell surface associated	5.22E-15	3.927286776
ALDH1A1	aldehyde dehydrogenase 1 family, member A1	3.29E-28	3.883302864
FOXA1	forkhead box A1	1.05E-54	3.872321151
DOCK10	dedicator of cytokinesis 10	1.32E-07	3.865119296
ADGRF4	adhesion G protein-coupled receptor F4	1.69E-16	3.81351502
LOC101927481	uncharacterized LOC101927481	2.65E-07	3.804819541

MYEOV	myeloma overexpressed	1.68E-27	3.796811624
GABRP	gamma-aminobutyric acid (GABA) A receptor, pi	1.49E-44	3.750625114
CABLES1	Cdk5 and Abl enzyme substrate 1	1.27E-27	3.684030232
MAL2	mal, T-cell differentiation protein 2 (gene/pseudogene)	4.19E-39	3.628079306
SAMSN1	SAM domain, SH3 domain and nuclear localization signals 1	3.4E-08	3.53872555
MTBP	MDM2 binding protein	2.01E-26	3.536326627
DNER	delta/notch-like EGF repeat containing	1.38E-39	3.529761982
OLR1	oxidized low density lipoprotein (lectin-like) receptor 1	1.8E-09	3.452287209
RTL1	retrotransposon-like 1	1.89E-08	3.445503451
RBPMS	RNA binding protein with multiple splicing	1.63E-27	3.357234354
TNFRSF19	tumor necrosis factor receptor superfamily, member 19	9.81E-11	3.287615671
KIAA1456	KIAA1456	2.43E-06	3.282493401
PTGES	prostaglandin E synthase	2.36E-43	3.275723158
IL1RN	interleukin 1 receptor antagonist	3.94E-27	3.24144319
UBE2V2	ubiquitin-conjugating enzyme E2 variant 2	7.98E-25	3.162006537
ETV5	ets variant 5	5.5E-21	3.150478939
RAET1E	retinoic acid early transcript 1E	4.43E-10	3.144542064
TMEM158	transmembrane protein 158 (gene/pseudogene)	1.05E-07	3.12554077
DUSP4	dual specificity phosphatase 4	1.2E-22	3.113136071

Table S3. PCR primers used for RT-PCR and QMSP

	Forward primer	Fluorescent probe	Reverse primer
HOPX (RT-PCR	TCAACAAGGTCGACAAGCAC	N/A	TCTGTGACGGATCTGCACTC
HOPX-β	TTTGGAGAGGGTTTTAAAGCG	FAM-CGGAGATAGAAGGTCGTTTATCGGGGAGGTCG-TAMRA	AACAAACTTAACAAATCGCGAA
β-actin	TGGTGATGGAGGAGGTTTAGTAAGT	FAM-ACCACCACCCAACACACAATAACAAACACA-TAMRA	AACCAATAAAACCTACTCCTCCCTTAA
